# Artificial Intelligence in Dental Education: Opportunities and Challenges of Large Language Models and Multimodal Foundation Models

**DOI:** 10.2196/52346

**Published:** 2024-09-27

**Authors:** Daniel Claman, Emre Sezgin

**Affiliations:** 1 Pediatric Dentistry, Nationwide Children’s Hospital, Columbus, OH, United States; 2 Department of Pediatrics, The Ohio State University College of Medicine, Columbus, OH, United States; 3 Center for Biobehavioral Health, The Abigail Wexner Research Institute at Nationwide Children’s Hospital, 700, Children’s Drive, Columbus, OH, 43205, United States, 1 6147223179

**Keywords:** artificial intelligence, large language models, dental education, GPT, ChatGPT, periodontal health, AI, LLM, LLMs, chatbot, natural language, generative pretrained transformer, innovation, technology, large language model

## Abstract

Instructional and clinical technologies have been transforming dental education. With the emergence of artificial intelligence (AI), the opportunities of using AI in education has increased. With the recent advancement of generative AI, large language models (LLMs) and foundation models gained attention with their capabilities in natural language understanding and generation as well as combining multiple types of data, such as text, images, and audio. A common example has been ChatGPT, which is based on a powerful LLM—the GPT model. This paper discusses the potential benefits and challenges of incorporating LLMs in dental education, focusing on periodontal charting with a use case to outline capabilities of LLMs. LLMs can provide personalized feedback, generate case scenarios, and create educational content to contribute to the quality of dental education. However, challenges, limitations, and risks exist, including bias and inaccuracy in the content created, privacy and security concerns, and the risk of overreliance. With guidance and oversight, and by effectively and ethically integrating LLMs, dental education can incorporate engaging and personalized learning experiences for students toward readiness for real-life clinical practice.

## Introduction

In recent years, dental education has experienced a significant transformation, driven by the rapid evolution of technology [1-3]. Dentistry faculties and educators have recognized the potential of these advancements to enhance the learning experience and guide patient care and have actively integrated them into their curricula [4]. Therefore, this has led to a change in the state of dental education in dental schools, focusing on the incorporation of technology to foster a more effective, engaging, and innovative learning environment. Specifically, the emergence of artificial intelligence (AI) has created a broader impact [1].

Dentistry has always been a highly specialized field, requiring a combination of theoretical knowledge, practical skills, and clinical acumen. Dental faculty have traditionally employed a combination of lectures, seminars, laboratory work, and supervised clinical practice to deliver a comprehensive educational experience to dental students. However, the advent of cutting-edge technology and AI has created new possibilities for improving the quality of education as well as the practice to better prepare future dental professionals [1,5,6]. By leveraging these technological advancements, dental educators are able to create more interactive and personalized learning experiences. Virtual reality, for instance, allows students to immerse themselves in realistic clinical scenarios, enhancing their understanding of complex dental procedures and techniques [7]. Similarly, haptic devices and 3D printing enable the development of accurate dental models, facilitating hands-on practice and improving students’ dexterity and confidence in performing intricate procedures [8]. Finally, the addition of advanced medical charting (eg, integrated electronic medical records or voice-activated periodontal charting) to clinical practice has required the dental school faculty to instruct on how best to use technology to provide safe clinical care when in practice [9-11]. Beyond all, AI has been perceived to improve operations, innovation, and practices in dental education at multiple levels [1].

## Transformation in Education and Technology With Large Language Models

As dentistry continues to evolve with the integration of advanced technologies, AI has emerged as a powerful tool with new potentials to improve dental education. One such innovation, which has been highly communicated recently, is large language models (LLMs). An LLM is a type of AI model designed to conceptualize and generate human-like text based on large amounts of data [12]. These models are trained on vast amounts of text from various sources online, enabling them to generate contextually relevant responses, summaries, translations, and more. LLMs have been argued to potentially transform various domains, including education, by providing personalized learning experiences and assisting in content creation [13]. Further ahead, multimodal foundation models (FM) are similar large-scale AI models which are pretrained on extensive data, enabling them to conceptualize and generate image and audio, in addition to the text [14].

Currently well-known LLMs and FMs, such as GPT (OpenAI) [15], LaMDA and PaLM (Google) [16], and LLaMA (Meta) [17], have shown potential in medical education and practice, including problem-solving, question and answering, summarization, and content creation [13,18,19]. Especially in dental education, it may provide innovative methods to enhance the learning experience for dental students. Personalized learning could be one, as these models can be used to create unique experiences for students by generating custom learning materials based on their individual needs, preferences, and learning styles [20,21]. In addition, LLMs and FMs can be used for content creation, where it can create educational content such as quizzes, assessments, and lesson plans which in turn can help educators save time and improve the quality of their teaching materials [20]. Therefore, it is important to explore the application of LLMs and FMs in dental education. In this perspective, to take a glimpse at applications in dental education, we share a use case of periodontal charting, and highlight major opportunities and challenges associated with AI implementation.

## Use Case: Periodontal Charting

### Overview

Periodontal charting, an important component of dental practice and clinical care, involves the systematic recording of information related to a patient’s periodontal health, such as probing depths, gingival recession, clinical attachment levels, and the presence of bleeding or suppuration. Accurate periodontal charting is essential for diagnosis, treatment planning, and monitoring the progress of periodontal therapy. Periodontal health is an important part of the dental school curriculum, and ultimately a significant component of clinical practice. Dental students are asked to complete numerous competency examinations on the assessment and treatment of periodontal disease. Additionally, periodontal assessment and treatment is a critical component of dental licensure examinations. Integrating generative models (eg, LLMs and FMs) into the teaching and learning process of periodontal charting offers several opportunities to improve students’ understanding and mastery of this important clinical skill [22].

One major opportunity is to provide personalized feedback and guidance. By inputting the students’ charting data and observations, a model can analyze the information, compare it with established guidelines and best practices [23], and generate tailored feedback. This feedback can highlight errors, suggest improvements, and reinforce correct techniques, supporting students in refining their charting skills and enhancing their clinical decision-making capabilities. Another opportunity is the creation of realistic case scenarios and simulations. LLMs and FMs can generate a wide range of patient cases with varying periodontal conditions, enabling students to practice periodontal charting in diverse clinical contexts. This exposure to a multitude of cases can foster deeper comprehension of the underlying principles and techniques, and better prepare students for real-life clinical practice.

However, there are also challenges associated with the integration of these models into dental education. One primary concern is the potential for biases or inaccuracies in the AI-generated content. As these models rely on the data they have been trained on, they may inadvertently reproduce erroneous information or reflect biases present in their training data [18]. To mitigate this issue, it is crucial for educators to carefully review and validate the content generated, ensuring that it aligns with current evidence-based practices and professional standards. Yet, validation might be challenging with tools such as ChatGPT, which do not provide the reference to the source of the information presented. Another challenge is the potential overreliance on AI by students, which may lead to a decreased emphasis on critical thinking and problem-solving skills [24]. To address this concern, educators must keep a balance between leveraging the benefits of AI and fostering independent learning. Encouraging students to critically evaluate the feedback and suggestions provided by AI can promote the development of essential clinical reasoning skills [24,25].

To demonstrate the capability of these generative AI (LLM or FM) models in dental education, we present a series of examples of LLM or FM applications as a periodontal charting educational support mechanism.

### Example 1: Periodontal Charting Feedback

In this example, a student enters a prompt to obtain feedback about performed periodontal charting. The prompt and response are presented in Textbox 1. We used the GPT-4 model (via ChatGPT application) [26] to generate this example in March 2023.

Textbox 1. **Periodontal charting prompt and response by GPT-4.**

Prompt:

*I performed periodontal charting on a patient and recorded the following data for tooth #3:*

- *Probing depths: 2 mm (mesial), 3 mm (buccal), 2 mm (distal)*
- *Gingival recession: 0 mm (mesial), 1 mm (buccal), 0 mm (distal)*
- *Bleeding on probing: absent (mesial), present (buccal), absent (distal)*
- *Suppuration: absent*

Response:

*Based on the information provided, it seems that the periodontal charting for tooth #3 is fairly comprehensive. The probing depths, gingival recession, bleeding on probing, and suppuration have all been recorded, which are important aspects of periodontal charting.*

*However, there are a few aspects that could be improved or clarified for a more complete assessment:*

1. *Six-point probing: To provide a comprehensive picture of the periodontal health around tooth #3, it's important to record probing depths for all six points (mesial-buccal, mid-buccal, distal-buccal, mesial-lingual, mid-lingual, and distal-lingual) rather than just three points (mesial, buccal, and distal). Consider adding the missing data for the lingual side of the tooth.*
2. *Consistency in recording: Ensure that all aspects of the charting (probing depths, gingival recession, bleeding on probing, and suppuration) are documented for each of the six points, if applicable. This will provide a more accurate representation of the periodontal health of tooth #3.*
3. *Mobility and furcation involvement: While not mentioned in the provided data, it is also essential to assess and record tooth mobility and furcation involvement, if present. These factors can have a significant impact on the periodontal prognosis and treatment planning.*
4. *Use of standard notation: Make sure to use standard notation when recording periodontal charting data. This will help to ensure that the information is clear and easily understood by other dental professionals who may be involved in the patient’s care.*

*Overall, the charting seems to be accurate for the data provided, but additional details are necessary for a complete periodontal assessment of tooth #3.*

### Example 2: Periodontal Disease Classification

**Figure 1.**
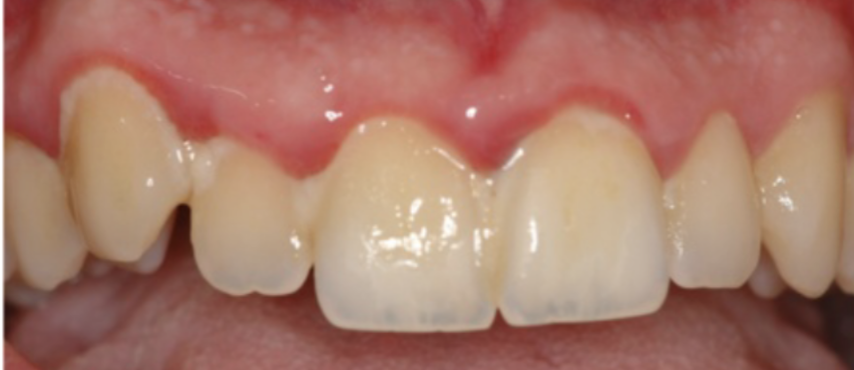
Image of upper jaw acute gingivitis (reproduced from Cafiero et al [27], which is published under Creative Commons Attribution 4.0 International License [28])

In this example, a student enters a prompt to obtain feedback about periodontal disease assessment. The prompt and response are presented in Textbox 2 (AI-based periodontal health assessment from Figure 1 [27]). The image was captioned as “Upper jaw acute gingivitis in a non-smoker 26-year-old male patient. Abundant plaque deposit is visible on the surfaces of the teeth” [27]. We used the GPT-4 model (via ChatGPT application) [26] to generate this example in January 2024.

Textbox 2. **Artifical intelligence–based periodontal health assessment.**

Prompt:

*What can you say about the periodontal health of these teeth?*

Response:

*Based on the provided image, there are several clinical signs indicative of periodontal health issues:*

*Gingival Inflammation: The gums appear to be red and swollen, particularly around the necks of the teeth. This is suggestive of gingival inflammation, which could be due to gingivitis or the initial stages of periodontitis.*

*Gingival Recession: There seems to be some recession of the gums, especially noticeable around the left central incisor. This could indicate a loss of periodontal attachment.*

*Plaque Accumulation: There are visible yellowish deposits near the gum line of the teeth, which likely represent plaque. Plaque accumulation is a primary factor in the development of both gingivitis and periodontitis.*

*Tooth Discoloration: There is also evidence of tooth discoloration, which may be related to dietary habits, oral hygiene practices, or possibly the presence of calculus (tartar), which is hardened plaque that can form both above and below the gum line. Gingival Margin Contours: The contour of the gingival margin appears to be irregular, which can be a sign of periodontal disease progression.*

*It's important to note that a definitive diagnosis would require a full periodontal examination, including periodontal probing to measure pocket depths, assessment of bleeding on probing, mobility testing, and possibly radiographs to evaluate bone levels. This image provides a snapshot that suggests the presence of periodontal issues, but a comprehensive evaluation by a dental professional is necessary for an accurate diagnosis and to formulate an appropriate treatment plan.*

### Example 3: Estimating Periodontal Pocket Depth

**Figure 2.**
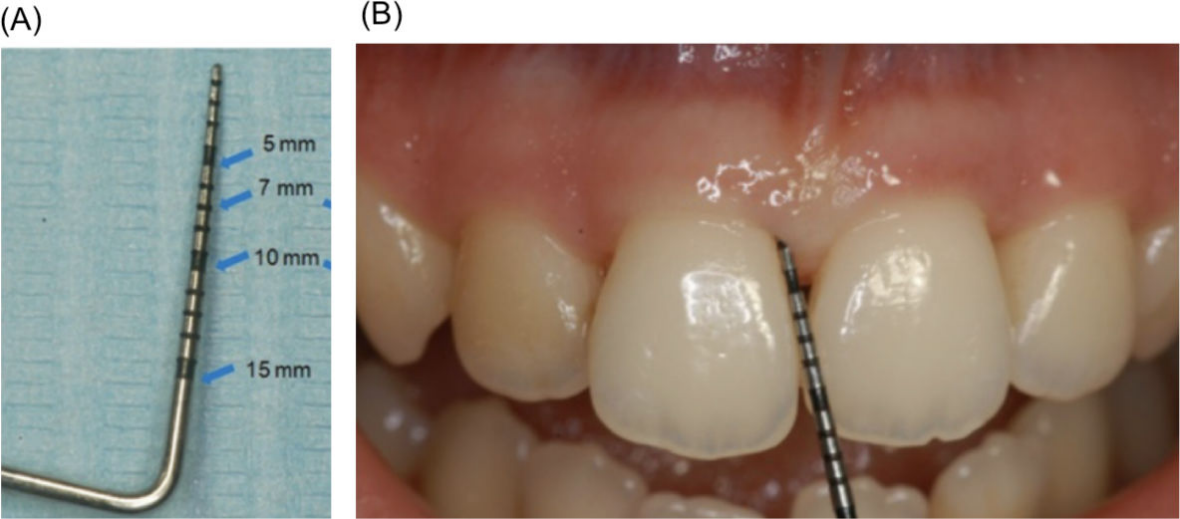
(A) Image of a periodontal probe; (B) image of a periodontal probe inserted into the sulcus (reproduced from Cafiero et al [27] which is published under Creative Commons Attribution 4.0 International License [28]).

In this example, a student enters a prompt to obtain feedback about the periodontal pocket depth (PPD) estimations. The prompt and response are presented in Textbox 3 (AI-based PPD assessment from Figure 2 [27]). The image was captioned as “A periodontal probe is inserted into the sulcus and in a parallel position with respect to the long axis of the tooth. The physiological value of PPD is considered to be ≤3 mm. PPD allows an immediate evaluation of diseased sites” (Textbox 3 and Figure 2) [27]. We used the GPT-4 model (via ChatGPT application) [26] to generate this example in January 2024.

The response provided by GPT-4 (as a multimodal FM) demonstrates a natural language understanding and image recognition for periodontal charting and questions. The readers should note that these examples do not provide the validity or accuracy of the model but rather a demonstration of its capability. However, in terms of the accuracy of exemplified cases, the response by GPT-4 is in line with general dental knowledge and practices.

Textbox 3. **Artificial intelligence–based periodontal pocket depth (PPD) assessment.**

Prompt:

*Here is the probe for your reference (* *Figure 2A* *). Now estimate the PPD in the next image (* *Figure 2B* *).*

Response:

*Now, with the reference image of the periodontal probe provided, I can give an estimate of the periodontal pocket depth (PPD) in this image. The probe’s tip appears to be between the 3 mm and 4 mm marks, as it does not reach the first black band (which I assume, based on common probe designs, indicates 5 mm). It is not clear how much of the probe is within the sulcus due to the angle of the photo and the overlap of the tooth. However, it seems that the probe may be inserted approximately 3 mm into the sulcus. Therefore, the estimated PPD in this image appears to be about 3 mm, which is within the normal physiological range. It's important to note that this is an estimation and that in clinical practice, measurements should be taken from multiple angles and at several points around each tooth for accuracy.*

In example 1, GPT-4 offers a critique of the data entered by the student. The response identifies areas where the charting is accurate and comprehensive and suggests areas where the charting could be improved or clarified. The feedback highlights the importance of 6-point probing, consistency in recording, mobility and furcation involvement, and the use of standard notation. These aspects are essential for dental students to learn and incorporate into their practice, as they will help to document an accurate and comprehensive periodontal assessment that will be used to enhance patient care. The suggestions for improvement, such as including readings from the lingual aspect of the tooth and ensuring consistency in recording, are valid. From an educational standpoint, the response offers a structured and informative critique that could be beneficial for dental students. The feedback emphasizes the importance of thorough periodontal assessments, which is essential for optimal patient care and treatment planning. Additionally, the response encourages the use of standard notation, which ensures clear communication among dental professionals.

In example 2, GPT-4 offers an evaluation of the given clinical image. The response evaluates the image, providing clinical details present on the image. The feedback highlights the presence of inflammation, plaque accumulation around the gingival margin, and discoloration of the teeth. This evaluation can be helpful in guiding dental students on what to clinically evaluate for periodontal and dental health and can demonstrate examples with analysis of healthy and unhealthy gingiva and dentition. There is a tendency for the model to extrapolate clinical findings from a simple image evaluation; however, the model does caution that a clinical examination should be completed to properly diagnose and evaluate. This response, generally, can be helpful in guiding dental students to properly evaluate and examine patients in the clinical setting.

In example 3, GPT-4 offers an evaluation of the depth indicated on the periodontal probing instrument. The feedback indicates the correct reading, and the appropriate justification for this analysis, while also providing information about common probes and how the probe indicators are arranged. This is helpful for dental students to understand common instrumentation and how to properly read these instruments in the clinical setting. From an educational standpoint, the response offers information on instrumentation from which students can learn. The feedback appropriately evaluates the information, gives context, and indicates the need for a full examination to corroborate findings.

## Challenges and Considerations

While the LLM and FM responses provide valuable feedback and highlight areas for improvement within the given use case on periodontal charting, it is essential to consider the potential challenges, risks and limitations associated with this approach on a broader scale.

Publicly available generative AI tools (such as GPT, LaMDA, and LLaMA) are not trained specifically on the dental domain. As probabilistic models, they predict how likely a particular sequence is to occur in the language based on a training data set and they reflect this. Therefore, they may generate different contents for very similar questions depending on the structure of the questions (Textbox 4). This inconsistency could also be the case with repetition of the same prompts with the same model [29]. This may not be ideal for standardization of the educational contents. In some occasions, it may create misleading, biased, or incorrect results, such as responses irrelevant to the question or prompt or responses note based on existing data for the prompt [18]. It is important to be aware that the information generated by the AI may contain such inaccuracies or biases, necessitating a thorough review, experiments, and creation of safeguards or operational guidelines by educators or clinicians to ensure alignment with evidence-based practices and ethical principles [30]. In addition, fine-tuning and advanced prompt engineering (eg, chain-of-thought or in-context learning) could be adopted to mitigate risks [31,32]. Unlike LLMs, FMs are multimodal and more versatile but may require significant fine-tuning with complex domain-specific data to mitigate the risk of generating generalized content that may not align with specialized dental knowledge or practice standards. This adjustment is crucial to tailor the outputs of FMs to reflect up-to-date clinical guidelines and diagnostic criteria specific to dentistry.

Textbox 4. **Differences in basic question answering (created by GPT-4 in March 2023). Authors asked GPT-4 to explain periodontal charting in 2 different question forms. The explanations in both responses are slightly different. Whereas response 1 defines periodontal charting as a dental diagnostic and monitoring tool, response 2 defines this as a procedure. Similarly, response 1 addresses the measuring tool as a “specialized probe,” whereas response 2 addresses this as a “periodontal probe.”**

Prompt 1: *What is periodontal charting?*

Response 1: *Periodontal charting is a dental diagnostic and monitoring tool used by dentists and dental hygienists to evaluate the health of a patient’s gums and teeth. During periodontal charting, the dental professional measures the depth of the space between the gum line and the tooth root, known as the periodontal pocket, using a specialized probe…*

Prompt 2: *Tell me about periodontal charting.*

Response 2: *Periodontal charting is a procedure performed by dental professionals to evaluate the health of a patient’s gums and teeth. It involves measuring the depth of the spaces between the gum line and the tooth root, known as periodontal pockets, using a periodontal probe…*

Regardless of the level of accuracy achieved by technical improvement, while adopting these tools, educators and students must be encouraged to critically assess the feedback provided by the AI to develop their clinical reasoning and problem-solving skills, rather than solely relying on the model’s output. Otherwise, there is a risk that students might become overly reliant on AI for decision-making, potentially undermining the development of their independent clinical judgment and manual skills. Similarly, the limitation of the models on detailed tasks, such as critical appraisal of literature, may further contribute to adverse outcomes, where educators consider adapting teaching and assessment methods to leverage AI’s benefits while mitigating risks such as academic dishonesty [21].

It is crucial to design educational programs that balance the use of AI with traditional hands-on and problem-solving training to ensure that students remain adept at both using technology and performing without it. This practice may further necessitate cultural and contextual specific considerations for dental practices in diverse environments, regarding regional differences in dental conditions, treatment preferences, and public health guidelines.

Furthermore, privacy and security of personal health information (PHI) are important to consider. Dental education often involves the use of patient data, including medical histories, diagnostic images, and clinical findings. When using LLMs and FMs in this setting, it is essential to ensure that PHI is not included and that the use of AI has been discussed and approved by the institutions in which they are being used. The entered information (including text and image) should be stripped of any identifying information (and images should be checked for not violating copyright laws) before being input into the AI model to prevent potential privacy breaches, especially with publicly accessible LLM and FMs, which are loosely governed or regulated. These models, as a dental education tool, ideally should be hosted on secure platforms with robust encryption and access controls to prevent unauthorized access and data breaches. Some institutions may provide secure cloud services via compliant service providers (eg, Microsoft Azure, Amazon Web Services, and Google), which may ensure a more private ecosystem for AI use. Various regulations govern the handling of PHI and the use in health care, such as the Health Insurance Portability and Accountability Act in the United States and the General Data Protection Regulation in the European Union [33]. These regulations set forth strict requirements for the management of PHI, including data privacy, security, and patient rights. In addition, dental education institutions using such AI models must ensure compliance with the relevant regulations in their jurisdiction to avoid legal repercussions and maintain the trust of patients and the dental community (eg, the recently proposed California AI accountability act necessitates transparency by requiring agencies to disclose interaction with AI and to conduct risk assessment before AI adoption) [34]. Table 1 outlines current challenges and strategies to address them in dental education.

**Table 1.** Strategies to address challenges with LLMs^a^ and FMs^b^ in dental education.

Category and strategy		Details
**Bias and inaccuracy mitigation**		
	Specialized training data sets and knowledge base	Use data sets compiled from a diverse range of dental texts, research papers, and case studies to train the LLM or FM or for use as part of the knowledge base, ensuring they cover various dental specialties and scenarios.
	Continuous clinical validation	Regularly validate LLM or FM outputs against current dental practices and standards by engaging with dental boards or professional groups.
	Domain-specific fine-tuning and guided prompt engineering	Work with dental faculty and practicing dentists to tailor the LLM or FM outputs to reflect up-to-date clinical guidelines and diagnostic criteria. In addition, use guided prompt engineering and alternative approaches (eg, chain-of-thought) to improve outputs.
**Operational guidelines**		
	Curriculum integration guidelines	Develop specific guidelines on how LLM or FM integrates into different parts of the dental curriculum, such as diagnostics, treatment planning, and patient communication.
	Professional oversight	Set up a committee of dental professionals to oversee the implementation and use of LLM or FM, ensuring alignment with educational outcomes and clinical accuracy.
**Enhancing student interaction**		
	Simulation-based learning	Incorporate LLM or FM into simulation settings where students can interact with AI^c^ to diagnose and treat virtual patients, enhancing their practical skills without risk.
	Reflective practice sessions	Facilitate sessions where students reflect on the AI’s suggestions compared to standard treatment protocols, promoting critical thinking and decision-making skills.
**Privacy and security**		
	Scenario-based training	Train students and staff on handling personal health information through scenario-based exercises, ensuring they understand how to manage data securely when using LLM or FM in dental settings.
	Enhanced encryption for dental data	Implement higher levels of encryption and security measures for platforms hosting dental data to ensure compliance and safeguard against breaches.
**Regulatory compliance**		
	Tailored compliance workshops	Hold workshops focused on the specific legal requirements related to using LLM or FM with PHI in the dental field, such as Health Insurance Portability and Accountability Act in the United States and General Data Protection Regulation in Europe.
	Ethical use guidelines	Develop ethical guidelines that address the nuances of using AI in dental training and practice, including issues of patient consent and AI transparency.
**Feedback and continuous improvement**		
	Feedback system for clinical use	Establish a structured feedback system where dental students and professionals can report inaccuracies or ethical concerns with AI outputs, facilitating continuous improvement.

a LLM: large language model.

b FM: foundation model.

c AI: artificial intelligence.

## Conclusions

The integration of LLMs and FMs into dental education holds promising opportunities for improving the quality of education and better preparing future dental professionals. By navigating the challenges and leveraging the potential benefits, dental educators can create more interactive, personalized, and innovative learning experiences that effectively prepare students for the complex and evolving world of dental practice. These considerations are also applicable for patient education and self-care practices as well. Considering the accessibility of these models to the public, educational considerations could be further expanded for patient education. Future works are suggested on gathering empirical evidence for the feasibility and utility of LLMs or FMs, including alternative prompt engineering approaches, fine-tuned custom model testing, user testing, cost-benefit analysis, and expanding AI guidelines for including generative AI use in dental education.

## Abbreviations

AI: artificial intelligence
FM: foundation model
LLM: large language model
PHI: personal health information
PPD: periodontal pocket depth
